# Supplementary material for: A spatially discretized convolutional neural mass model for studying meso-scale spatio-temporal transformations in the rat hippocampus
Source: Res Sq. 2026 Apr 13:rs.3.rs-9306977. Preprint. [Version 1] doi: 10.21203/rs.3.rs-9306977/v1 (PMC13105126; doi:10.21203/rs.3.rs-9306977/v1)
Supplement: 1 [file NIHPPRS9306977V1-supplement-1.pdf]

Appendix A Supplementary Figures

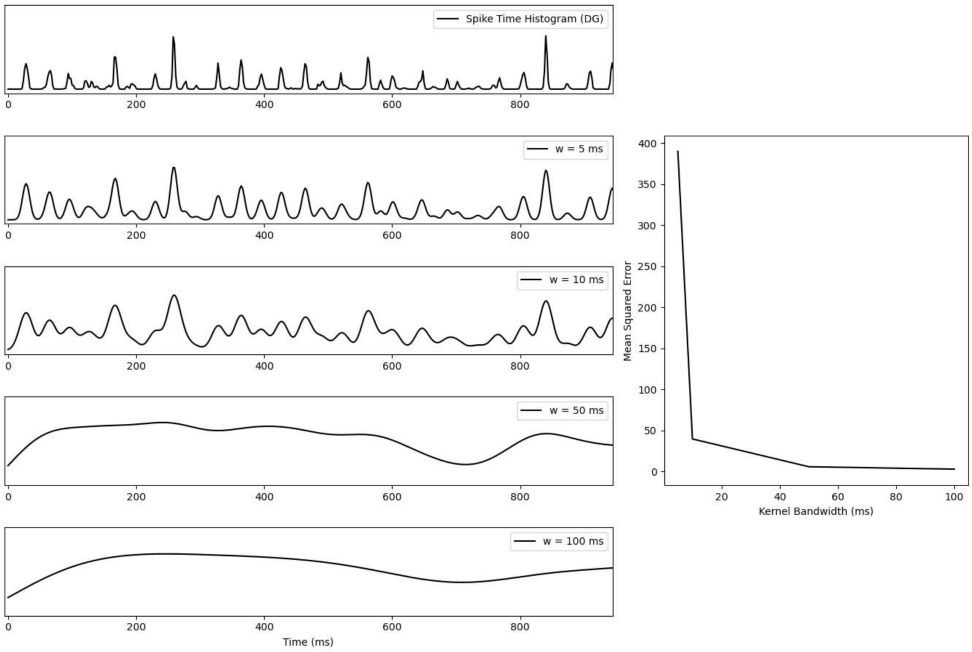

Fig. A1 Effect of kernel bandwidth. (Left) The top plot shows an example of the raw spike time histogram of input DG spiking activity (from the CA3 neural mass closest to the center) while the plots below illustrate the effect of convolving the spike time histogram with Gaussian kernels of varying bandwidths (w). The vertical axis is normalized for all plots and given in arbitrary units. Note, the larger the kernel bandwidth, the more the signal approaches a constant flat line. (Right) The mean squared error of the CNMM's estimate is shown as a function of kernel bandwidth. In general, the larger the kernel bandwidth, the more accurate the model is. However, increasing kernel bandwidth indefinitely removes meaningful features of the original signal.

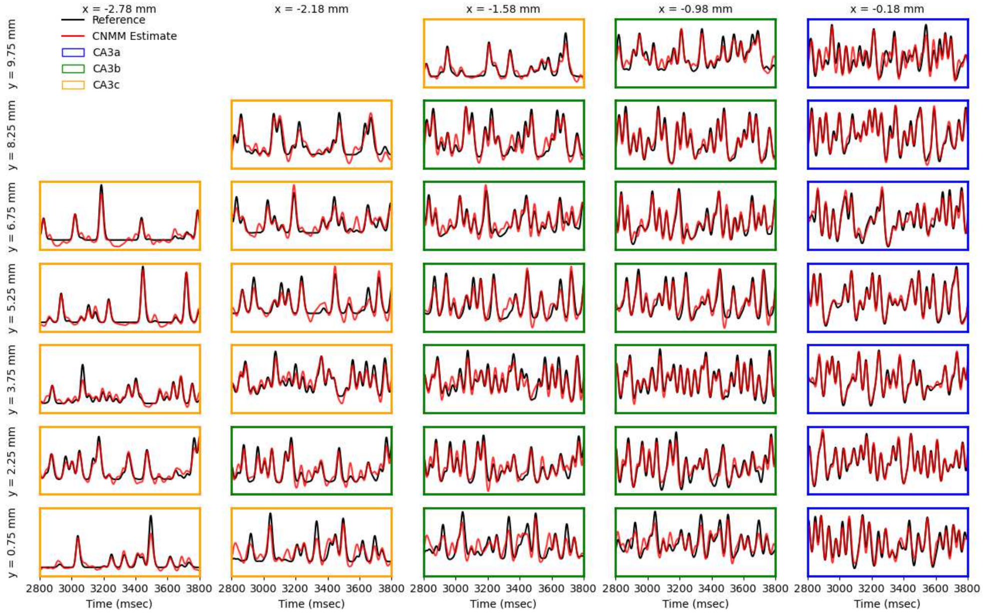

Fig. A2 CNMM spike density estimates due to random MEC activity. Same as Fig. 7 but input-output data were recorded from a LSM simulation involving random input MEC spiking activity rather than the original rat exploration task (driven by grid cells) to evaluate the generalization capability of the CNMM. The CNMM metaparameters and weights were from the previous training that originally optimized to rat exploration data. Traces are normalized and are therefore shown in arbitrary units.

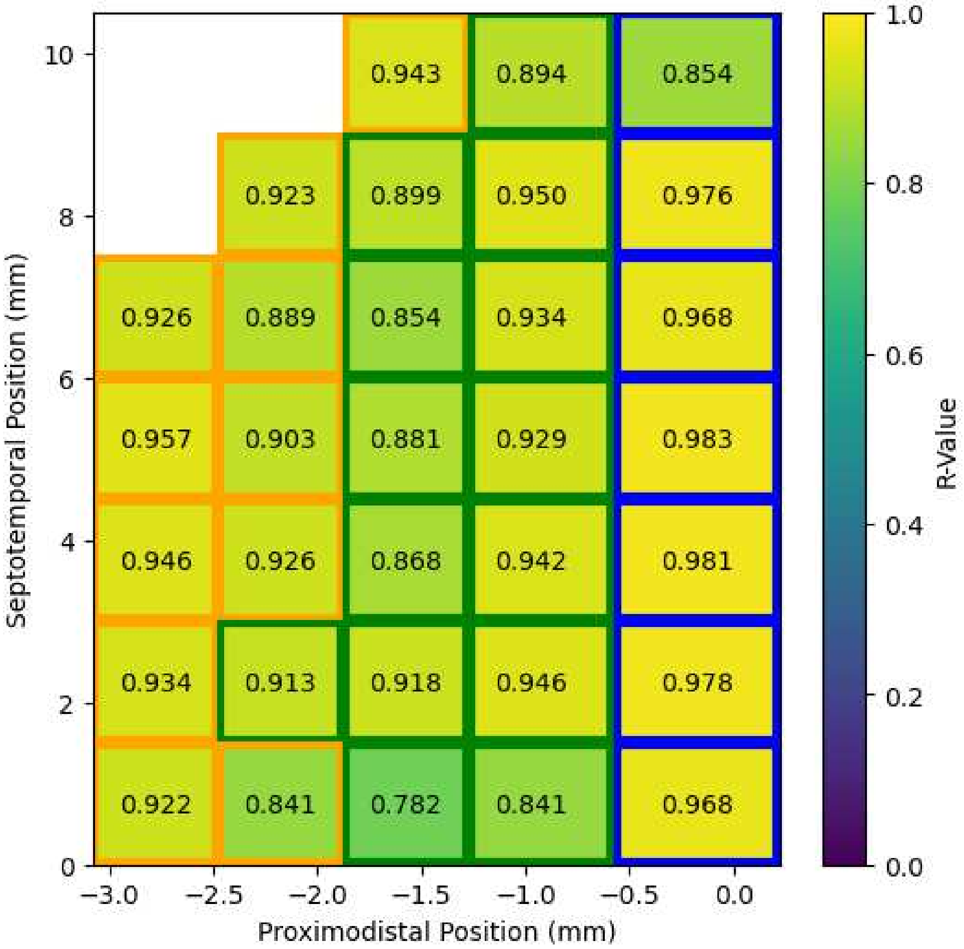

Fig. A3 CNMM spike density prediction accuracy due to random MEC activity. The R values for each of the 32 neural masses in Fig. A2 are shown.

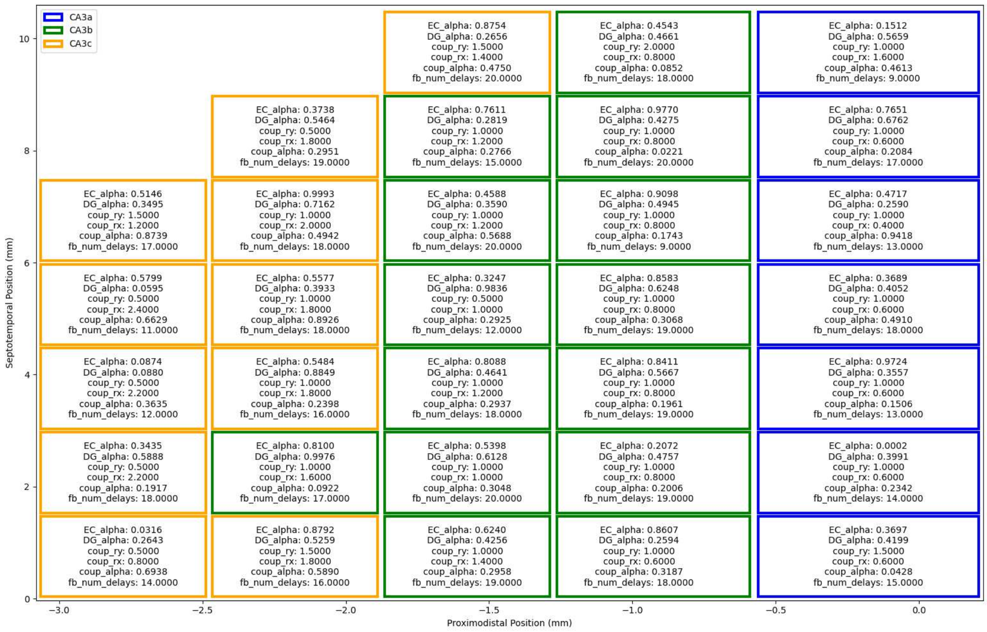

Fig. A4 CNMM optimized metaparameters for spike density estimation. The values of the optimized metaparameters for spike density estimation are shown across a two-dimensional map of CA3 for the 32 chosen neural masses.

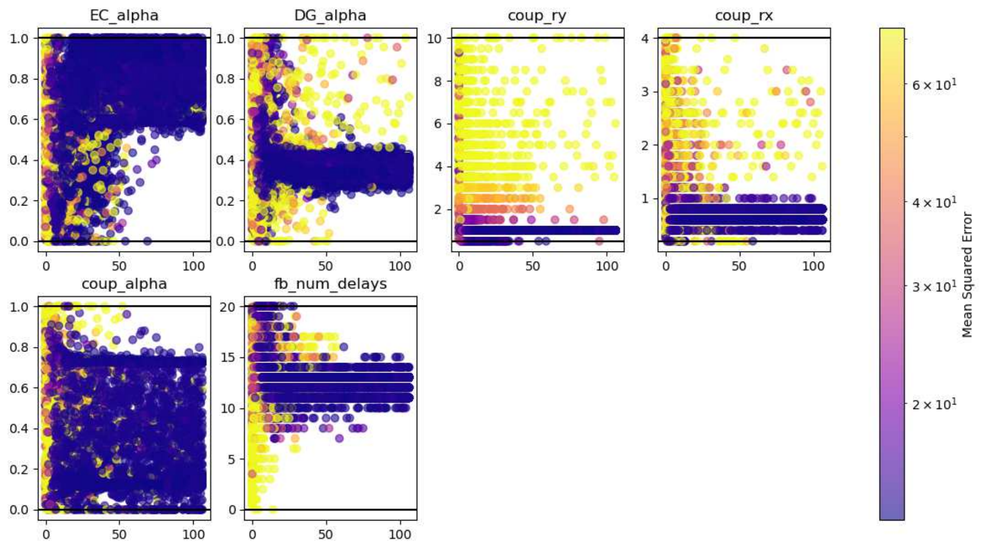

Fig. A5 Exploration of metaparameter space during PSO. An example of the evolution of the metaparameters during Particle Swarm Optimization (PSO) is shown. The x-axis represents the iteration number. Each dot represents the value of a metaparameter for an individual particle out of a population of 200. The color of each dot represents the loss (mean squared error) associated with the particle. Horizontal black lines represent the boundaries of the metaparameter values. Alpha and fb_num_delays are unitless while the ry and rx have units of mm. From visual inspection, the CNMM is most sensitive to the alpha value of the DG kernel, the r values of the coupling kernel, and the fb_num_delays value as demonstrated by the tight region of dense purple (i.e., low error) expressed by those metaparameters toward the later iterations.
